# Biomechanical Adjustments of the Basketball Jump Shot Performed Over Differently High Opponents

**DOI:** 10.2478/hukin-2022-0049

**Published:** 2022-09-08

**Authors:** Tim Kambič, Filip Stepišnik Krašovec, Frane Erčulj, Igor Štirn

**Affiliations:** 1 Department of Research and Education, General Hospital Murska Sobota, Murska Sobota, Slovenia; 2 Faculty of Sport, University of Ljubljana, Ljubljana, Slovenia; 3 Sopron Basketball Club, Sopron, Hungary

**Keywords:** kinematics, jump shot, shooting angle, efficiency, basketball performance

## Abstract

Biomechanical adjustments of the jump shot in presence of an opponent and their associations with shooting efficiency remain to be determined in elite basketball. The aim of this research was to examine the selected biomechanical determinants of the jump shot when shooting over opponents of different height. Nineteen elite basketball players, age 22 ± 3 years, performed three trials of 20 basketball shots in a crossover, randomised manner: over an obstacle of the height of standing reach (RH), over reach height with additional 20 cm (RH+20 cm), over reach height with additional 40 cm (RH+40 cm), and the maximum height jump shot without an obstacle (JS_max_). Jump height, the ball entry angle, and shooting efficiency were measured on each trial. Jump height when shooting over RH+40 cm was significantly higher than RH+20 cm (+0.022 m, p = 0.030) and RH (+0.023 m, p = 0.029). Similarly, the ball entry angle was greater at RH+40 cm compared to RH (+7.19 °, p < 0.001) and RH+20 cm (+2.90°, p < 0.001). In contrast, shooting efficiency decreased significantly when shooting over RH+40 cm compared to RH (-10.79%, p = 0.048) and RH+20 cm (-8.95%, p = 0.015). We recorded the highest jump height (0.35 ± 0.08m, p < 0.001) and the lowest angle of entry (39.16 ± 1.19°, p < 0.001) when participants performed JS_max_. Shooting over higher opponents should be prioritised in training to significantly improve shooting efficiency. Future research is needed to determine additional potential biomechanical determinants of a successful jump shot in elite basketball.

## Introduction

Basketball is a team sport with the aim to score more points than the opponent by shooting the ball through a 46 cm circular rim (Okazaki et al., 2015). The basketball shot is the only way to score and is one of the most important technical elements in basketball, executed with either the one-legged lay-up under the rim or the two-legged standing or jump shot from distance (Podmenik et al., 2012).

A successful shot is determined by three main factors of the ball trajectory during the release phase: the vertical displacement of the ball, the horizontal displacement and the ball velocity (Miller and Bartlett, 1996). Previous research has mainly suggested that higher shooting efficiency can be achieved when the basketball jump shot is performed at its highest point in the air, and when using a lower shooting velocity and a release ball angle between 44° and 52° (Miller and Bartlett, 1996; Rojas et al., 2000). However, there are several additional factors that influence successful field goal (Okazaki et al., 2015), including shooting distance (Miller and Bartlett, 1996, 1993; Okazaki and Rodacki, 2012), body position (posture) during ball release (Knudson, 1993), presence of an opponent guarding the shot (Rojas et al., 2000), the angle of release and entry of the basketball (Miller and Bartlett, 1993, 1996), field of view (Oudejans et al., 2002), and other movements (e.g., dribbling, passing) prior to shooting (Okazaki et al., 2015; Southard and Miracle, 1993).

Previous research has shown that changes in shooting distance alter the height and the angle of ball release, e.g., a longer shooting distance results in a lower jump height and thus a lower ball release height (Miller and Bartlett, 1993, 1996; Okazaki and Rodacki, 2012). These factors decrease shooting efficiency (Okazaki et al., 2015). All of the above studies were conducted without the presence of a defender guarding the shot. However, one of the few studies applied the presence of an opponent during shooting and reported that the angle and height of the released ball increased when shooting over a 1.95 m opponent compared to shooting without the presence of the opponent (Rojas et al., 2000). The presence of the opponent also resulted in greater shooting angles of the shoulders and arms at release, probably due to a faster release of the ball when avoiding the block. On the other hand, compared to shooting without an opponent, there was no significant difference in the height of the centre of gravity during jumping (Rojas et al., 2000).

The presence of an opponent at early stages of the jump shot may also obscure the view of the basketball rim. It has been shown that late vision of the basketball rim (vision obscured until the last +/-350 ms before the ball release) can be as effective as shooting with full vision throughout the jump shot execution, while late vision (vision obscured during the last +/-350 ms before the ball release) severely impaired shot efficiency (Oudejans et al., 2002).

Despite the elite level of developed skills in professional basketball, players such as Stephen Curry are always searching new ways to improve their jump shot (Cohen, 2018). Previously, it was demonstrated that the shooting angle and shooting velocity may alter shooting efficiency (Khlifa et al., 2012, 2013; Miller and Bartlett, 1996; Rojas, et al., 2000). Studies have shown increased shooting efficiency when players increase their shooting angle (Khlifa et al., 2012, 2013), and when shooting over opponents (Rojas et al., 2000). Since only one study has examined biomechanical adjustments while shooting over smaller opponents (Rojas et al., 2000), there is still a need to further investigate biomechanical adjustments of the jump shot when performed over taller opponents. Therefore, the aim of our study was to further investigate biomechanical adjustments of the jump shot performed over opponents of different height and their associations with shooting efficiency in professional basketball. We hypothesised that shooting over higher opponents would increase the entry angle of the basketball and the height of the jump shot, although it would decrease shooting efficiency when compared to shooting over smaller opponents.

## Methods

### Participants

Nineteen elite basketball players, age [mean (SD)] 22 (3) years, height 190.80 (0.96) cm, body mass 86.50 (1.37) kg, volunteered to participate in the study. All participants played in the first or the second Slovenian basketball league and had 13 (1) years of basketball experience. To ensure homogeneity of the sample, only point guards and shooting guards were included in the sample because they make an easier biomechanical adjustment to their shooting kinematics, and they shoot more frequently from longer distances compared to players of other positions (Miller and Bartlett, 1996). The exclusion criteria were any recent injury or illness within six months. Prior to enrolment in the study, all athletes were verbally informed and signed written consent to participate in the study. The study was approved by the Board of Sport Ethics at the Faculty of Sport, University of Ljubljana, and was conducted in accordance with the Declaration of Helsinki and the American College of Sports Medicine guidelines for the use of Human Participants.

### Measurements

Body height and mass were measured to the nearest 0.01 cm and 0.01 kg, respectively, using a commercial scale and a stadiometer (SECA, Hamburg, Germany). Ground reaction forces during shooting trials and CMJs were measured using the Kistler 9286A force plate (Kistler Instruments AG , Winterthur, Switzerland), while shooting angles were measured using 94Fifty® Smart Basketball (InfoMotion Sports Technologies Inc., Massachusetts, USA). The smart basketball recorded real-time kinematic information during the basketball session (e.g., forces applied to the basketball during the shot, shooting angles, shooting time, angular velocity of basketball rotation) and enabled direct feedback to the measurer. This device proved to be reliable (± 1 degree shooting angle error) and showed excellent validity compared to the Dartfish system (Cronbach's alpha = 0.998) (Abdelrasoul et al., 2015) and to the Kinovea software at 100 Hz video capture (significant correlation coefficient for the ball entry angle = 0.98 and velocity = 0.96, both *p* < 0.05) (Rupčić et al., 2016). In professional basketball, the ball is usually released in the highest point of the jump shot (Okazaki et al., 2015), thus, the data obtained from both devices were not synchronised.

### Design and procedures

#### Design

This study was designed as a crossover, randomised controlled study. To assess the shooting angle (ball entry angle), shooting efficiency, and shot jump height, participants performed three randomly assigned sets consisting of 20 shots per set: shooting over the obstacle of standing reach height (RH), over an obstacle 20 cm higher than RH (RH+20 cm), and over an obstacle 40 cm higher than RH (RH+40 cm). For additional assessment of jump height, participants performed three countermovement jumps (CMJs) with and without an arm swing followed by three repetitions of maximum height jump shots without an obstacle.

#### Procedures

**Figure 1:**
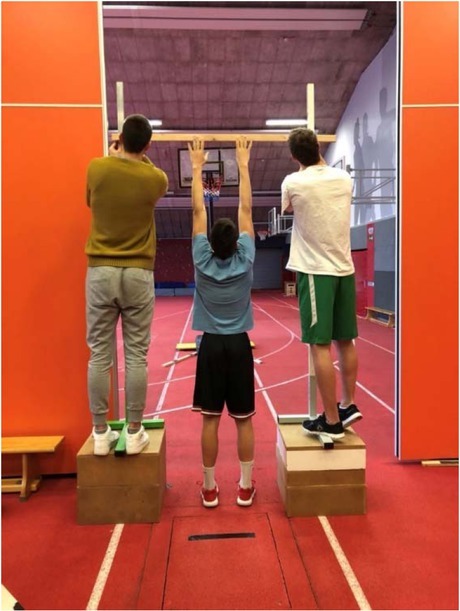
Measurement of reachable height of the participant

All measurements were performed in the Laboratory of Kinesiology at the Faculty of Sport, University of Ljubljana. Participants were advised to rest and avoid any moderate-to-vigorous physical activity, including basketball and resistance training, as well as to avoid ingesting caffeine on the morning of the measurements. Each participant visited our facilities only once. Prior to the warm-up, we measured each athlete's maximal vertical standing reach height (Figure 1).

The warm-up lasted 8 minutes and consisted of common basketball movements. After the warm-up, each participant performed three shots from a distance of 5.40 m and 60 cm from the obstacle (imitating a defender) in a brief familiarization session. Before each shooting trial, we determined the baseline force (weighted) of each participant while standing on a force plate with hand holding the basketball. Time zero was determined by instructing athletes to stand still for 3-5 s prior to performing a basketball jump shot. During the test trial, participants were randomly assigned to perform 20 jump shots over three different obstacle heights: standing reach height (RH), standing reach height additionally raised by 20 cm (RH+20 cm), and 40 cm (RH+40 cm). In the additional shooting trial, participants were asked to perform 20 maximal height jump shots without obstacles (JS_max_). There was a five-minute rest interval between each shooting trial. In the final measurement, participants performed three CMJs with and without arms assistance (swing) during the take-off phase, with a two-minute rest interval between the two CMJ attempts.

### Statistical analysis

Numerical variables are presented as means (standard error of the mean) unless otherwise stated. Normality of distribution, homogeneity of variances, and sphericity were tested using the Shapiro-Wilk test, the Levene's test, and the Mauchly's test, respectively. Differences between shooting trials (RH, RH+20, RH+40 and JS_max_) were calculated using repeated measures analysis of variance (ANOVA), with Bonferroni correction for pairwise comparisons for normally distributed variables and equal variances; the Friedman test was used for pairwise comparisons. Data were analysed using the IBM SPSS 22 statistical package for Windows (SPSS, Chicago, Illinois, USA), with alpha level set a priori to 0.05.

## Results

**Figure 2a–c.**
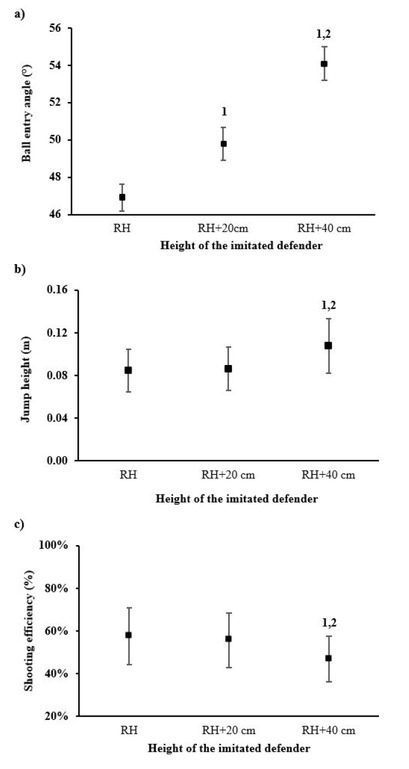
Differences in ball entry angles (a), jump height (b) and shooting efficiency (c) while shooting over different heights of the imitated defender. RH-reaching height, 1-significantly different from RH, 2-significantly different from RH+20 cm

There was a significant difference in jump height (*p* < 0.001), ball entry angles (*p* = 0.020), and shooting efficiency (*p* = 0.031) between shooting trials. Shooting over RH+40 cm resulted in a significantly higher jump height compared to RH+20 cm (+0.022 m, *p* = 0.030) and RH (+0.023 m, *p* = 0.029) (Figure 2a). Similarly, the angle of entry differed significantly across all three shooting trials (*p* < 0.001), with the highest difference obtained between RH and RH+40 cm (+7.19°, *p* < 0.001) and the lowest between RH and RH+20 cm (+2.90°, *p* < 0.001) (Figure 2b). On the contrary, shooting efficiency decreased significantly when shooting over higher obstacles (*p* = 0.031) (Figure 2c).

Shooting efficiency decreased significantly when shooting over RH+40 cm compared to RH (10.79%, *p* = 0.048) and RH+20 cm (-8.95%, *p* = 0.015). When performing JS_max_, we obtained the significantly highest value of jump shot height (0.350 [0.08] m, *p* < 0.001) and the lowest angle of entry (39.16 [1.19]°, *p* < 0.001). Finally, the average CMJ heights with and without an arm swing were 0.470 (0.054) m and 0.388 (0.045) m, respectively.

## Discussion

Our study showed that shooting over higher obstacles, imitating blocking by a defensive player, resulted in higher jumping height and an increased ball entry angle, but lower shooting efficiency. The largest differences in measured variables were obtained between RH, RH+40 and JS_max_. When performing the maximum jump shot without an obstacle (0.350 [0.08] m), participants achieved 74.43% and 90.18% of the height of the maximum CMJ with and without arm assistance, respectively.

The latter results are consistent with previous reports measuring maximum CMJ height in basketball point guards, which varied from 44.8 to 52.6 cm in average and elite point guards, respectively (Ziv and Lidor, 2010). The efficiency in our study was little lower (<60%, Figure 2c) compared to the efficiency (62.0%) when shooting without an opponent 4.6 m from the basket (Okazaki and Rodacki, 2012). Apart from the fact that players performed shooting over an obstacle, the shooting distance in our study was also longer (5.40 m), thus the results are not surprising.

Previous studies have shown that shooting biomechanics and efficiency are largely dependent on jump height, distance to the rim, and presence of an opponent (Okazaki and Rodacki, 2012; Okazaki et al., 2015; Rojas et al., 2000). One of the studies showed that shooting over a 1.95 m opponent resulted in a significantly increased ball release angle (47.0 [1.7]°) compared to shooting without the presence of an opponent (44.7 [2.3]°), as a result of the higher height of the ball release (Rojas et al., 2000). Similar results were obtained in our study, where the ball entry angle when shooting over an obstacle of standing reach height was 46.4 (0.72)°. We found even larger entry angles with increasing height of the obstacle in the RH+20 and RH+40 trials. Although the kinematics of ball release was not measured, it can be postulated that the increase in the angle of entry was strongly dependent on the increased height of ball release, at least for shooting over RH and RH+20cm, while no significant difference was found for the jumps when the height was very low (< 9 cm).

In our study, the height of the shooting obstacle limited shooting efficiency when shooting over RH+20 cm and RH+40 cm. The vision was not obscured in our experiment, thus it seems that the additional height of the obstacle, but not the altered visual control (due to the view of the basket obscured by the defender) was the main reason for the reduced efficiency. Indeed, some previous reports showed no significant difference in efficiency when shooting with full vision to the basket or with late vision at the release phase of the shot (Oudejans et al., 2002).

We identified a few limitations of our study. Firstly, we did not measure ball entry angles and shooting efficiency when shooting without obstacles. Secondly, the accuracy of the data collection could be improved by filming the execution of the jump shot. This would serve as an external validation of the signals obtained from the smart basketball and the force plate. Lastly, our study only provided selected biomechanical features (e.g., ball entry angle and jump shot height), thus, it would be interesting in future studies to investigate wrist, shoulder, and hip release angles, as well as an effect of visual control (e.g., gaze behaviour), as previously reported (de Oliveira et al., 2008; Štirn et al., 2019). Nevertheless, this study provides new insights into the biomechanics of shooting over higher opponents, which is a very common situation in the game.

Our study showed that shooting success largely depends on the opponent's block height, which may alter the biomechanics of the basketball jump shot. Our findings can be applied in basketball training to improve shooting form and efficiency when shooting over opponents of different height. However, future research is needed to investigate other biomechanical variables (the distance to the block, the dynamic (moving) block, etc.) that may contribute to adaptations of the basketball jump shot when shooting over opponents of different height.
